# User-centered design and the development of patient decision aids: protocol for a systematic review

**DOI:** 10.1186/2046-4053-4-11

**Published:** 2015-01-26

**Authors:** Holly O Witteman, Selma Chipenda Dansokho, Heather Colquhoun, Angela Coulter, Michèle Dugas, Angela Fagerlin, Anik MC Giguere, Sholom Glouberman, Lynne Haslett, Aubri Hoffman, Noah Ivers, France Légaré, Jean Légaré, Carrie Levin, Karli Lopez, Victor M Montori, Thierry Provencher, Jean-Sébastien Renaud, Kerri Sparling, Dawn Stacey, Gratianne Vaisson, Robert J Volk, William Witteman

**Affiliations:** Office of Education and Continuing Professional Development, Faculty of Medicine, Laval University, Pavillon Ferdinand-Vandry 2881, Quebec City, QC G1V 0A6 Canada; Department of Family and Emergency Medicine, Laval University, 1050 avenue de la Médecine, Pavillon Ferdinand-Vandry 4617, Quebec City, QC G1V 0A6 Canada; Research Centre of the Centre Hospitalier Universitaire de Québec, 1 rue de l’Espinay, Hôpital Saint François d’Assise D6-727, Quebec City, QC G1L 3L5 Canada; Occupational Science and Occupational Therapy, Rehabilitation Sciences Building, 160-500 University Ave, Toronto, ON M5G 1V7 Canada; Health Services Research Unit, Nuffield Department of Population Health, University of Oxford, Old Road Campus, Oxford, OX3 7LF UK; Center for Bioethics and Social Sciences in Medicine, Division of General Medicine, Department of Internal Medicine, University of Michigan, 2800 Plymouth Road, Building 16, Rm. 421W, Ann Arbor, MI 48109-2800 USA; VA Health Services Research and Development Center of Excellence, VA Ann Arbor Healthcare System, Ann Arbor, MI USA; Quebec Centre for Excellence in Aging, Research Centre of the CHU de Quebec, St-Sacrement Hospital, Local L2-08, 1050 chemin Sainte-Foy, Quebec City, QC G1S 4L8 Canada; Patients Canada, 3560 Bathurst Street, Toronto, ON M6A 2E1 Canada; East End Community Health Centre, 1619 Queen St.E, Toronto, ON M4L1G4 Canada; Department of Community and Family Medicine, The Dartmouth Institute for Health Policy and Clinical Practice, Geisel School of Medicine at Dartmouth, 46 Centerra Parkway (HB3250), Lebanon, NH USA; Family Practice Health Centre and Institute for Health Systems Solutions and Virtual Care, Women’s College Hospital, 77 Grenville Street 4th Floor, Toronto, ON M5S 1B3 Canada; Department of Family and Community Medicine, University of Toronto, 500 University Avenue, 5th Floor, Toronto, ON M5G 1V7 Canada; Arthritis Alliance of Canada, 403 rue des Érables, Neuville, Québec G0A 2R0 Canada; Informed Medical Decisions Foundation, Healthwise, Inc, 40 Court Street, Boston, MA 02108 USA; 6811 Baxter Terrace Cir, Anchorage, AK 99504 USA; Knowledge and Evaluation Research Unit, Mayo Clinic, Rochester, MN 55905 USA; Six Until Me, 5600 Post Rd, Unit 228, East Greenwich, RI 02818 USA; School of Nursing, University of Ottawa, 451 Smyth Road (RGN 1118), Ottawa, ON K1H 8M5 Canada; Ottawa Hospital Research Institute, 725 Parkdale Ave, Ottawa, ON K1Y 4E9 Canada; Department of Health Services Research, The University of Texas MD Anderson Cancer Center, P.O. Box 301402, Houston, TX 77230-1444 USA

**Keywords:** Patient decision aids, Decision support, Shared decision-making, Patient education, Counseling, User-centered design, Human-centered design, Patient partnership, Stakeholder engagement, Implementation, Knowledge translation, Patient-centered care

## Abstract

**Background:**

Providing patient-centered care requires that patients partner in their personal health-care decisions to the full extent desired. Patient decision aids facilitate processes of shared decision-making between patients and their clinicians by presenting relevant scientific information in balanced, understandable ways, helping clarify patients’ goals, and guiding decision-making processes. Although international standards stipulate that patients and clinicians should be involved in decision aid development, little is known about how such involvement currently occurs, let alone best practices. This systematic review consisting of three interlinked subreviews seeks to describe current practices of user involvement in the development of patient decision aids, compare these to practices of user-centered design, and identify promising strategies.

**Methods/design:**

A research team that includes patient and clinician representatives, decision aid developers, and systematic review method experts will guide this review according to the Cochrane Handbook and PRISMA reporting guidelines. A medical librarian will hand search key references and use a peer-reviewed search strategy to search MEDLINE, EMBASE, PubMed, Web of Science, the Cochrane Library, the ACM library, IEEE Xplore, and Google Scholar. We will identify articles across all languages and years describing the development or evaluation of a patient decision aid, or the application of user-centered design or human-centered design to tools intended for patient use. Two independent reviewers will assess article eligibility and extract data into a matrix using a structured pilot-tested form based on a conceptual framework of user-centered design. We will synthesize evidence to describe how research teams have included users in their development process and compare these practices to user-centered design methods. If data permit, we will develop a measure of the user-centeredness of development processes and identify practices that are likely to be optimal.

**Discussion:**

This systematic review will provide evidence of current practices to inform approaches for involving patients and other stakeholders in the development of patient decision aids. We anticipate that the results will help move towards the establishment of best practices for the development of patient-centered tools and, in turn, help improve the experiences of people who face difficult health decisions.

**Systematic review registration:**

PROSPERO CRD42014013241

**Electronic supplementary material:**

The online version of this article (doi:10.1186/2046-4053-4-11) contains supplementary material, which is available to authorized users.

## Background

Patients are increasingly becoming involved in health research, not only as research participants but as partners with valuable expertise, perspectives, and insights for setting agendas, planning and carrying out projects, interpreting findings, and translating new knowledge to patient communities [1, 2]. Patient partnership in research teams is increasingly encouraged or required by funding organizations [3–6]. However, there are few empirically based best practices for research partnerships between patients, other stakeholders, and researchers.

The question of how to best involve patients in research is especially relevant in the development of patient decision aids. Patient decision aids are structured tools, often booklets or websites, that aim to provide unbiased, evidence-based information and guidance to patients making health decisions [7]. Unlike more general health education materials such as information leaflets, decision aids specifically support decision-making by making the decision explicit, providing balanced information on benefits and harms of options, and helping patients clarify what is most important in their own circumstances. They are intended to be used by patients to complement information and counseling from a health-care professional in the process of shared decision-making [8] and provide a means for clinicians and patients to collaboratively incorporate their expertise, insights, and views in order to make evidence-based health decisions that are aligned with patients’ preferences [9, 10].

The International Patient Decision Aid Standards (IPDAS) Collaboration stipulates that the development of a patient decision aid should follow a systematic process and should involve consultation with patients and clinicians. However, due to the lack of a robust evidence base from which to draw conclusions about best practices, practical guidance is minimal and vague [11–14]. Notably, only about half of patient decision aids included in the most recent Cochrane review of treatment and screening patient decision aids [7] reported having involved patients in their development process in some way [14].

Leaders in the field have offered insights based on their experiences of developing decision aids, including the importance of consulting with patients and other stakeholders [15–17], but there is little empirical evidence available about methods for putting patients at the center of the process. Accordingly, a recent update of the evidence base by the International Patient Decision Aid Standards Collaboration called for greater research into methods for patient involvement in decision aid development. Specifically, the relevant chapter in the updated standards states: “More guidance is needed to inform patient decision aid alpha- and beta-tests, including user-centered design methods […]. The process of designing the patient decision aid remains rather subjective” [13].

Our overall aim in this project is to ultimately improve the effectiveness, usability, and uptake of patient decision aids by identifying effective methods for involving patients and other stakeholders in their development. To accomplish this, we have identified three specific aims for this systematic review: (1) To describe how patients and other stakeholders, including clinicians, have and have not been involved in the development of patient decision aids. (2) To compare methods used for engaging patients and other stakeholders in decision aid development with methods of user-centered design. (3) To identify promising strategies for involving patients and other stakeholders in the development of patient decision aids.

### Conceptual framework

**Figure 1:**
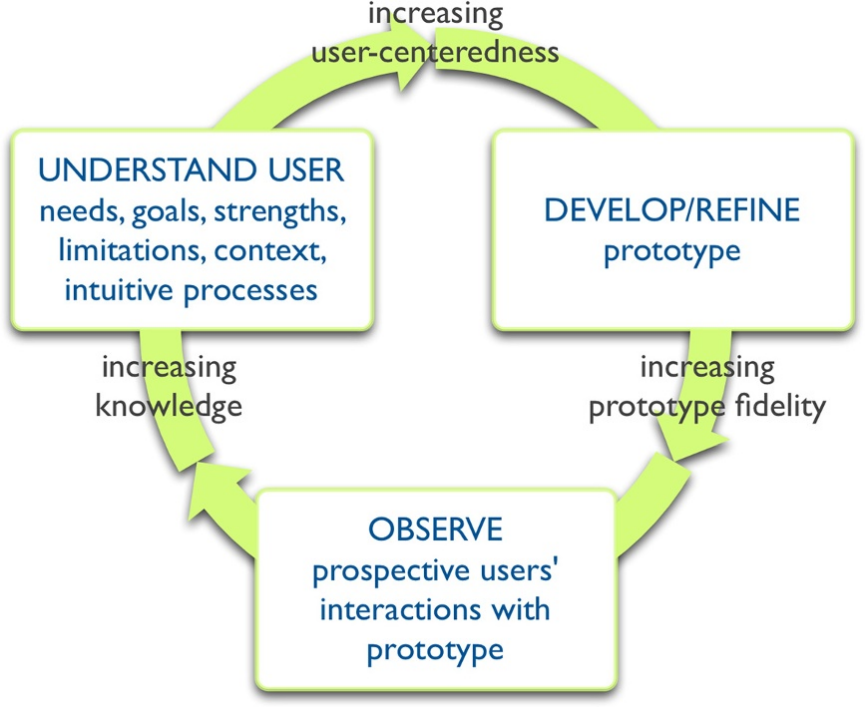
**Framework of user-centered design.**

To structure our research questions and data extraction plan, we will use a conceptual framework of user-centered design, a longstanding and proven framework and methodology for the development of products, services, and systems [18–22] that has yet to be widely applied in the domain of health care [23–27]. User-centered design is a highly iterative method for optimizing the user experience—and thus the effectiveness—of a system, service, or product [18, 28–30]. In this framework, a user is any person who interacts with (in other words, “uses”) the system, service, or product for some purpose. Figure 1 shows a visual depiction of user-centered design, distilled from seminal work in the field of Human Factors [18, 20, 22, 31–33]. The term user-centered design is often used interchangeably with human-centered design [22].

This framework rests on the idea that a system, service, or product is most likely to fulfill user needs when its development process is based on iterative cycles in which potential users are consulted early and often. In the case of patient decision aids, lack of a fully iterative feedback loop may result in decision aids that are not optimized to meet people’s needs. When users are not able to critique a design until it is far along in the development process, it may be too late to make certain types of changes given time and cost constraints. Research in the field of user-centered design in non-health contexts offers some guidance regarding, for example, how many potential users might need to participate in a software development process to ensure that a product is fully usable [34–36]. However, it is unknown whether or not these findings are applicable to patient-oriented tools such as decision aids and whether or not the framework might serve as a useful guide to optimize the development of such tools.

This conceptual framework will serve two purposes in this review. Its primary purpose will be to structure our data extraction process. We will ensure that our data extraction form captures each element in the framework. Its secondary purpose will be to help generate and structure hypotheses for exploratory analyses.

## Methods/design

**Figure 2:**
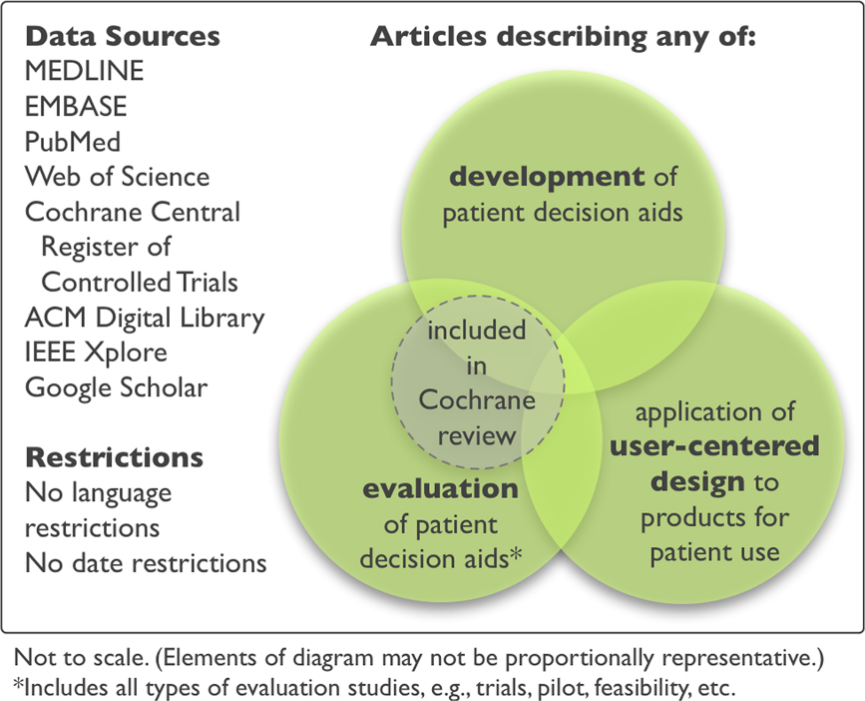
**Search strategy overview.**

This systematic review will be guided by the Cochrane Handbook and reported according to the PRISMA guidelines. This review comprises three related and potentially overlapping subreviews (see Figure 2) and will accordingly address the three following research questions: When developing patient decision aids, (1) how are patients and other stakeholders currently involved, (2) how do these practices compare to those within user-centered design, and (3) which practices are associated with better outcomes, for example, increased knowledge scores, indications of better usability, or improved clarity about one’s values relevant to a decision?

### Inclusion and exclusion criteria

We will include three potentially overlapping groups of articles (see Figure 2) in order to conduct our planned syntheses and comparisons.

The first group of articles will be those that describe the development of a decision aid intended to support a patient’s health decision or the decision of a surrogate regarding a patient. We will use data from these articles in addressing all three research questions. To be eligible for inclusion in this group, articles must explicitly describe at least one step in the development process of a patient decision aid. We will exclude articles that do not meet this criterion.

The second group of articles will be those that explicitly describe the application of user/human-centered design in the development of patient-oriented tools. We will use data from these articles to address research questions 2 and 3. These articles may describe patient-oriented tools that go beyond decision aids, e.g., tools for education, social support, or self-management. This includes health management tools used by members of the public who are not necessarily active patients in the health-care system. We will exclude related articles about applying user/human-centered design to tools intended for use solely by clinicians.

The third group of articles will be those that describe the evaluation of a patient decision aid. We will use data from these articles to address research question 3. Articles about evaluation of decision aids may describe preliminary testing phases, feasibility and acceptability studies, pilot studies, randomized controlled trials, or other types of evaluations of a developed patient decision aid. This project is expanding upon an existing systematic review, namely, the Cochrane review of decision aids for screening and treatment decisions [7], which is led by a member of our research team (DS). This review will necessarily include all articles within the Cochrane review, as well as others. These other articles will largely describe quantitative studies, but we will also include some qualitative studies or studies that include both qualitative and quantitative results, for example, those that describe the evaluation of the usability of a prototype patient decision aid. Evaluation studies may focus on the efficacy of a decision aid (e.g., capacity to increase patient knowledge or decrease decisional conflict within the context of a formal study), its actual or potential implementation (e.g., was it successfully integrated into routine care, does a feasibility study suggest it is implementable), and/or its effectiveness (e.g., does it show desired effects in real-world conditions). We will exclude articles that do not meet this criterion.

Articles will be screened for inclusion by two independent reviewers who will first examine titles and abstracts to assess potential relevance. All articles deemed potentially relevant based on their title and abstract will then be reviewed in full by two independent reviewers. Any discrepancies in inclusion and exclusion at any stage of this process will be addressed through regular discussions among the principal investigator, research staff, and medical librarian (HOW, SCD, MD, TP, GV, WW).

We will track articles’ inclusion and exclusion for reporting and will link together articles published separately about the same decision aid. This linking will account for the fact that it is common practice for research teams to publish a preliminary article describing the development and perhaps initial testing of a decision aid, followed by a second article one or more years later describing the evaluation of that decision aid.

### Literature search

We will perform a systematic literature search in MEDLINE, EMBASE, PubMed, Web of Science, the Cochrane Library, the ACM library, and IEEE Xplore. We also use Google Scholar for citation analysis of certain key papers, such as the 2006 International Patient Decision Aid Standards. An overview of our search strategy is shown in Figure 2.

We anticipate considerable overlap between our first and third groups, that is, articles describing the development of a patient decision aid and articles describing the evaluation of patient decision aids. For example, it is common for decision aid developers to publish an article describing the development and feasibility testing of a decision aid. Therefore, to identify articles in the first and third groups, we will seek articles in the union of these groups. We will search using the term “development” both as a free-text term and as a controlled-vocabulary term (MeSH, EMTREE), in conjunction with terms distinguishing the references we wanted from those describing biological development. We will also use other search terms such as usability and sets of terms such as test, study, or evaluation in close conjunction with terms like pilot or feasibility. To identify articles describing evaluation studies that were not randomized controlled trials, we will use a peer-reviewed, published search string that filters for observational studies [37]. We will search for articles in the intersection of any of these and an adapted version of the search string used to identify decision aids in the Cochrane review of decision aids [7]. See a sample search string in Additional file 1 that shows the entire strategy described above. We will also examine each article included in the Cochrane review of decision aids for screening or treatment decisions [7] and search its references to identify previous articles published by the authors that might have described the development process of the decision aid.

To identify articles in the second group (application of user-centered design to tools intended for patient use), we will search for the explicit use of the free-text terms “user-centred design”, “user-centered design”, “human-centred design”, or “human-centered design”, because for this group we will be seeking articles in which the authors clearly identify their development method as user/human-centered design.

Prior to conducting searches, our search strategy will be peer-reviewed by another medical librarian not on our study team.

### Data abstraction and validation

Data from each article will be abstracted by two independent, trained research team members using a standardized and pilot-tested data extraction form. The preliminary form will be based on our conceptual framework of user-centered design and relevant standards. Once we have established a preliminary form, we will conduct semi-structured consultations with approximately 12 key informants whom we believe can comment on our data extraction plan and potentially make suggestions for its improvement. These key informants may be researchers who have expertise in the area of development of patient-centered tools such as decision aids, or they may be patients or stakeholders whom we believe have a perspective not already represented on this project team. We will aim to consult with people who will bring a diverse set of perspectives and expertise. We will also pilot test the form with randomly selected articles from each of the three groups. We will iteratively revise the form, taking into account key informants’ comments and the results of pilot tests.

Once the form is finalized, pairs of data abstractors will independently extract data from all articles. Lack of agreements will be resolved through discussion until consensus is reached, either with the principal investigator and research staff (HOW, SCD, MD, TP, GV, WW), with the project’s steering committee (HOW, AF, AG, SG, FL, KS, RJV), or with the full team (all authors).

From all articles, we will extract key factors grounded in our conceptual framework of user-centered design, including: (i) whether patients and other stakeholders were involved in the development and, if yes, (ii) how they were defined (e.g., people who had previously faced the decision, people who might potentially face this decision, patients who were actually facing this decision, caregivers, clinicians who were not part of the research team), (iii) how were they identified and recruited, (iv) what information was collected about their needs and personal contexts, (v) how many were recruited, (vi) how often were patients and stakeholders consulted in the development process (i.e., were there iterative cycles of consultation, and, if yes, how many cycles), (vii) at what point(s) in the process were they involved (i.e., at the beginning during idea generation, once a fully developed prototype was prepared), (viii) what did their involvement consist of (i.e., were they observed interacting with the decision aid in a naturalistic fashion or were they asked to speculate on how they might use it), (ix) types of feedback sought (comprehensiveness or appropriateness of content, format, other), (x) how was their feedback incorporated into the design, and (xi) were members of potentially underserved populations involved (for example, patients with limited access to health care, people with lower health literacy, patients who have other health conditions that might complicate the decision under study, etc.). We will also record secondary data about whether or not each decision aid cited a relevant guideline, and the theory(ies) or framework(s) underpinning each decision aid. In addition, from articles describing the evaluation of a decision aid, we will extract information about the type of evaluation, specifically, whether or not outcomes such as feasibility, acceptability, and usability were reported, and if so, what metrics were used and what was reported according to those metrics. Any such additional evaluation data will be added to existing evaluation data from the Cochrane review of patient decision aids [7].

Throughout the data extraction process, research assistants will enter all data extracted into a data matrix structured according to our conceptual framework of user-centered design. In the matrix, we will match up articles describing the development of a patient decision aid with any evaluation data for that decision aid. We will track the source of each row in the matrix, i.e., whether it is a patient decision aid or a patient-oriented tool (for example, a self-management tool that is not oriented around decisions) developed with a user-centered design approach.

Because of the heterogeneity of reporting about development processes—namely, some research teams do not describe their processes at all, and, of those that do, the detail given varies considerably—and because we wish to ensure that we have understood each team’s work correctly, we will contact authors to review the data we have extracted about their study and collect any data that they did not or were unable to report in their publication. We will track which data were abstracted from the article and which were provided directly by authors.

### Quality assessment

The quality of each article will be appraised by two independent reviewers using adapted established quality criteria for scoring and appraising studies in a mixed method review [38]. We will track and record appraisal for reporting purposes.

### Evidence synthesis and analysis

To address our first research question (when developing patient decision aids, how are patients and other stakeholders currently involved?), we will assess and report frequency of use of different practices used in patient decision aid development, including the extent to which methods used in decision aid development align with methods of user-centered design by examining descriptive frequencies of practices within our conceptual framework. For example, we will determine the proportion of patient decision aids developed with involvement by patients who have previously faced the decision. This will allow us to develop an overall picture of practices used (or not used). If sufficient data exist for this purpose, we will also explore descriptively how practices may have changed over time.

To address our second research question (how do development practices for patient decision aids compare to those within user-centered design?), we will also assess and report the same descriptive frequencies for the development of other tools designed for patient use where authors of published reports explicitly described the development approach as user-centered design or human-centered design. We will explore potential differences by comparing and contrasting the development processes of patient decision aids with the development of other patient-centered tools explicitly employing user-centered design. For these comparisons, any patient decision aids that explicitly report using user-centered design will be treated separately, depending on the question addressed.

To address our third research question (which development practices are associated with better outcomes?), we will use a two-pronged analytic approach. First, in addition to examining descriptive frequencies for our first research question, if the data permit such development, we will also seek to develop a measure of patient- and stakeholder-centeredness of patient decision aid development processes. Second, if the data permit, we will also explore whether some strategies for patient involvement are associated with better outcomes, for example, increased knowledge scores, indications of better usability, or improved clarity about one’s values relevant to a decision. If it is possible to develop a measure as described above, we will use that measure as the independent variable in these latter analyses. Otherwise, we will conduct exploratory analyses using multiple independent variables selected primarily on the basis of their frequency of use. The primary outcomes for these analyses will be the same as those in the Cochrane review of patient decision aids, including patient knowledge and clarity about one’s values [7]. A secondary outcome of particular relevance to this review will be indications of usability. Given that this is a relatively new outcome in health research, the method of synthesis for this outcome is difficult to fully specify in advance, as it will depend on what is reported in the included studies. We anticipate synthesizing whether or not usability was reported at all, and if yes, metrics of ease of use (for example, the percentage of users rating a tool as easy to use), whether or not lists of usability problems were generated and addressed, and any reported validated measures of usability. This synthesis plan may be adapted according to reported outcomes in the identified articles.

## Discussion

With this systematic review, we aim to provide empirical evidence to help guide the inclusion of patients and other stakeholders in the development processes of patient decision aids with the ultimate goal of improving their effectiveness, usability, and uptake. The framework of user-centered design offers a robust and yet underused framework for examining current and potential methods for developing tools for patient use. By working to improve methods for involving patient partners and other stakeholders in the development process, we seek to help ensure that research responds to patient priorities and concerns and makes the best possible use of patients’ and other stakeholders’ time and expertise, as well as limited research funds. If data allow, this project may also contribute a measure of the user-centeredness of decision aid development that could be used to evaluate the quality of future patient decision aids’ development processes.

It is important to note that while this project will be conducted in the context of decision aid development, we anticipate that our findings may also apply more broadly to research involving development of other patient- or caregiver-oriented tools, services, and systems. Such other tools may be designed for purposes other than decision support, for example, patient education materials and self-management tools such as chronic disease management systems, applications for communication with health-care teams, and patient portals for electronic medical records. Development processes for these could also benefit significantly from optimal methods for patient and other stakeholder involvement. Because user-centered design is rooted in the field of human-computer interaction, we anticipate that our findings may be especially pertinent to those developing technology-oriented applications such as patient portals and mobile or online health applications. Providing this kind of guidance to improve the development processes of such tools, services, and systems may help increase their patient-centeredness.

## Electronic supplementary material

Additional file 1: **Sample search string (EMBASE.com Syntax).** (DOCX 13 KB)

## Abbreviations

ACM: Association for computing machinery
IEEE: Institute of Electrical and Electronics Engineers
IPDAS: International patient decision aid standards
PCORI: Patient-Centered Outcomes Research Institute
PRISMA: Preferred reporting items for systematic reviews and meta-analyses.
