# Supplementary material for: User-centered design and the development of patient decision aids: protocol for a systematic review
Source: Syst Rev. 2015 Jan 26;4(1):11. doi: 10.1186/2046-4053-4-11 (PMC4328638; doi:10.1186/2046-4053-4-11)
Supplement: Supplementary file 1 — Additional file 1: Sample search string (EMBASE.com Syntax). (DOCX 13 KB) [file 13643_2014_329_MOESM1_ESM.docx]

**Additional file 1: Sample Search String (EMBASE.com syntax)**

| #38 | #36 OR #37 |
| --- | --- |
| #37 | #7 AND #19 |
| #36 | #7 AND #35 |
| #35 | #20 OR #21 OR #22 OR #23 OR #24 OR #27 OR #28 OR #29 OR #30 OR #31 OR #32 OR #33 OR #34 |
| #34 | cross AND sectional NEXT/2 (study OR studies) |
| #33 | epidemiologic$ NEXT/2 (study OR studies) |
| #32 | observational NEXT/2 (study OR studies) |
| #31 | 'follow up' NEXT/2 (study OR studies) |
| #30 | 'case control' NEXT/2 (study OR studies) |
| #29 | cohort NEXT/2 (study OR studies) |
| #28 | 'cohort analysis'/exp |
| #27 | #25 NOT #26 |
| #26 | 'randomized controlled trials'/exp |
| #25 | 'prospective study'/exp |
| #24 | 'retrospective study'/exp |
| #23 | 'longitudinal study'/exp |
| #22 | 'family study'/exp |
| #21 | 'case control study'/exp |
| #20 | 'clinical study'/exp |
| #19 | #8 OR #11 OR #12 OR #13 OR #14 OR #15 OR #16 OR #17 OR #18 |
| #18 | patient* NEAR/3 (satisfact* OR opinion* OR knowledg* OR outcome* OR 'decisional conflict') |
| #17 | user NEAR/5 (stud* OR test* OR evaluat* OR experience) |
| #16 | usability |
| #15 | pilot NEXT/1 (test* OR stud* OR evaluat*) |
| #14 | acceptability |
| #13 | 'feasibility' NEAR/5 (test* OR stud* OR evaluat*) |
| #12 | evaluation |
| #11 | #9 NOT #10 |
| #10 | grow* |
| #9 | 'development'/exp OR 'development' |
| #8 | (user OR human) NEXT/2 (centered OR centred) |
| #7 | #1 OR #2 OR #3 OR #4 OR #5 OR #6 |
| #6 | informed NEAR/2 (choice* OR decision*) |
| #5 | 'shared decision making' |
| #4 | computer* NEXT/2 'decision making' |
| #3 | 'decision-making computer assisted'/exp |
| #2 | decision NEAR/3 (board* OR guide* OR counsel*) |
| #1 | (decision* OR decid*) NEAR/4 (support* OR aid* OR tool* OR material*) |
